# Supplementary material for: The diet of otters (Lutra lutra) on the Agri river system, one of the most important presence sites in Italy: a molecular approach
Source: PeerJ. 2020 Jul 29;8:e9606. doi: 10.7717/peerj.9606 (PMC7395603; doi:10.7717/peerj.9606)
Supplement: Supplemental Information 1 [file peerj-08-9606-s001.pdf]

Supplemental information

Valuation of primers

The 16Smam_1 (5'-CGGTTGGGGTGACCTCGGA-3) and 16Smam_2 (5'-GCTGTTATCCCTAGGGTAACT-3') primers (Tylor *et al.,* 1999) were tested to: I) assess their ability to bind and amplify the DNA sequences of the prey of the otter available in study area and II) checked (post-sequencing) if their amplification efficiency could influence the yield in terms of number of reads.

Firstly, using Geneious Primer 2019 v.2.1 (https://www.geneious.com), we performed multiple alignments of the primer set with the 16S mtDNA sequences (N = 390) of otter's potential prey in the study area (Caricato *et al.,* 2004), downloaded from National Center for Biotechnology Information (NCBI GenBank) nucleotide database (Table S1). Then, we revealed the number of mismatch between each primer and each sequence and we correlated the number of mismatches between the primer and the sequence assigned to a species with read count assigned to that species.

The primers bind the DNA of all considered potential otter's prey in the study area (fish and amphibian), with a mismatches between primer and prey sequence not higher than 4 (for 16Smam_1) and 1 (for 16Smam_2) nucleotide bases.

The correlation between the number of mismatches and the read count does not show a proportional trend between the variables, indeed, in examples, we did not reveal a decrease in read count due to the increase in the number of mismatch (Fig. S1 A and B).

The spectrum of variation, in terms of read count, which is highlighted at the level of 2 mismatches (for the primer forward, Fig. S1 A) and 0 mismatches (for the primer revers, Fig. S1 B) is always wider than the reads count values with higher mismatches.

Supplemental Table S1. List of the mitochondrial DNA sequences that include the 16S rRNA gene (partial or complete), downloaded from National Center for Biotechnology Information (NCBI) GenBank nucleotide database, with their accession number, corresponding organism, description, sequence length and topology. These sequences were used to performed multiple alignments with the primer 16Smam_1 and 16Smam_2 sequences, in Geneious Primer 2019 v.2.1 (https://www.geneious.com).

Accession number	Organism	Description	Sequence length	Topology
AF404839.1	*Acipenser sturio*	*Acipenser sturio 16S ribosomal RNA gene, partial sequence; mitochondrial gene for mitochondrial product*	515	linear
KP997216.1	*Acipenser sturio*	*Acipenser sturio mitochondrion, complete genome*	16555	circular
NC_027417.1	*Acipenser sturio*	*Acipenser sturio mitochondrion, complete genome*	16555	circular
JQ436546.1	*Alburnus alburnus*	*Alburnus alburnus isolate Alb1 16S ribosomal RNA gene, partial sequence; mitochondrial*	620	linear
AB239593.1	*Alburnus alburnus*	*Alburnus alburnus mitochondrial DNA, complete genome*	16605	circular
NC_008659.1	*Alburnus alburnus*	*Alburnus alburnus mitochondrion, complete genome*	16605	circular
AJ247063.1	*Alburnus alburnus*	*Alburnus alburnus partial mitochondrial 16S rRNA gene*	426	linear
KJ128688.1	*Alburnus alburnus*	*Alburnus alburnus voucher NRM:46979 16S ribosomal RNA gene, partial sequence; mitochondrial*	552	linear
EU853300.1	*Alburnus arborella*	*Alburnus arborella isolate AlbalbBO1 16S ribosomal RNA gene, partial sequence; mitochondrial*	538	linear
KF534726.1	*Alburnus chalcoides aralensis*	*Alburnus chalcoides aralensis mitochondrion, complete genome*	16604	circular
JX844591.1	*Alburnus istanbulensis*	*Alburnus istanbulensis mitochondrion, complete genome*	16612	circular
AP011281.1	*Alburnus mossulensis*	*Alburnus mossulensis mitochondrial DNA, complete genome*	16604	circular
MN122939.1	*Alosa fallax*	*Alosa fallax mitochondrion, partial genome*	16498	linear
EU552738.1	*Alosa fallax*	*Alosa fallax voucher H49 16S ribosomal RNA gene, partial sequence; mitochondrial*	542	linear

KC461223.1	*Alosa fallax lacustris*	*Alosa fallax lacustris voucher CL109 16S ribosomal RNA gene, partial sequence; mitochondrial*	563	linear
KY231825.1	*Ameiurus melas*	*Ameiurus melas isolate A_melas_002 16S ribosomal RNA gene, partial sequence; mitochondrial*	546	linear
LC198796.1	*Ameiurus melas*	*Ameiurus melas mitochondrial gene for 16S ribosomal RNA, partial sequence, isolation_source: aquarium water sample 3*	94	linear
LC198795.1	*Ameiurus melas*	*Ameiurus melas mitochondrial gene for 16S ribosomal RNA, partial sequence, isolation_source: laboratory tissue sample A_melas_002*	99	linear
KT804702.1	*Ameiurus melas*	*Ameiurus melas mitochondrion, complete genome*	16512	circular
DQ421877.1	*Ameiurus melas*	*Ameiurus melas voucher LSUBlk2 16S ribosomal RNA gene, partial sequence; mitochondrial*	501	linear
DQ421876.1	*Ameiurus melas*	*Ameiurus melas voucher MTMBL12 16S ribosomal RNA gene, partial sequence; mitochondrial*	501	linear
AJ247083.1	*Ameiurus melas*	*Ictalurus melas partial mitochondrial 16S rRNA gene*	374	linear
KX440971.1	*Anguilla anguilla*	*Anguilla anguilla 16S ribosomal RNA gene, partial sequence; mitochondrial*	243	linear
AP007233.1	*Anguilla anguilla*	*Anguilla anguilla mitochondrial DNA, complete genome*	16683	circular
U05965.1	*Aphanius fasciatus*	*Aphanius fasciatus 16S mitochondrial ribosomal RNA, mitochondrial gene, partial sequence*	439	linear
KY033528.1	*Aphanius fasciatus*	*Aphanius fasciatus voucher UFRJ<museum>:8076 16S ribosomal RNA gene, partial sequence; mitochondrial*	565	linear
KR476913.1	*Aspius aspius*	*Aspius aspius isolate Leuasp1 16S ribosomal RNA gene, partial sequence; mitochondrial*	511	linear
KR476832.1	*Aspius aspius*	*Aspius aspius isolate Leuasp2 16S ribosomal RNA gene, partial sequence; mitochondrial*	512	linear
DQ664301.1	*Aspius aspius*	*Aspius aspius isolate vis8 16S ribosomal RNA gene, partial sequence; mitochondrial*	524	linear

KJ128706.1	*Aspius aspius*	*Aspius aspius voucher NRM:47527 16S ribosomal RNA gene, partial sequence; mitochondrial*	553	linear
KJ128707.1	*Aspius aspius*	*Aspius aspius voucher NRM:54878 16S ribosomal RNA gene, partial sequence; mitochondrial*	553	linear
AB849085.1	*Atherina boyeri*	*Atherina boyeri mitochondrial gene for 16S rRNA, partial sequence, specimen_voucher: FRLM:37722*	502	linear
HM855098.1	*Atherina boyeri*	*Atherina boyeri voucher BOAlm1 16S ribosomal RNA gene, partial sequence; mitochondrial*	449	linear
HM855117.1	*Atherina boyeri*	*Atherina boyeri voucher BOTur2 16S ribosomal RNA gene, partial sequence; mitochondrial*	488	linear
HM855118.1	*Atherina boyeri*	*Atherina boyeri voucher BOTur3 16S ribosomal RNA gene, partial sequence; mitochondrial*	488	linear
HM855130.1	*Atherina boyeri*	*Atherina boyeri voucher NPCapZeb2 16S ribosomal RNA gene, partial sequence; mitochondrial*	447	linear
HM855125.1	*Atherina boyeri*	*Atherina boyeri voucher NPIs1 16S ribosomal RNA gene, partial sequence; mitochondrial*	446	linear
HM855136.1	*Atherina boyeri*	*Atherina boyeri voucher PLav3 16S ribosomal RNA gene, partial sequence; mitochondrial*	446	linear
HM855137.1	*Atherina boyeri*	*Atherina boyeri voucher PLav6 16S ribosomal RNA gene, partial sequence; mitochondrial*	446	linear
AY749073.1	*Atherina boyeri*	*Atherina boyeri voucher UPDBSG 0126 16S ribosomal RNA gene, partial sequence; mitochondrial*	490	linear
AY749074.1	*Atherina boyeri*	*Atherina boyeri voucher UPDBSG 0127 16S ribosomal RNA gene, partial sequence; mitochondrial*	485	linear
AY749075.1	*Atherina boyeri*	*Atherina boyeri voucher UPDBSG 0128 16S ribosomal RNA gene, partial sequence; mitochondrial*	486	linear
AF237604.1	*Austropotamobius pallipes*	*Austropotamobius pallipes F773 16S ribosomal RNA gene, partial sequence*	513	linear
KP205430.1	*Austropotamobius pallipes*	*Austropotamobius pallipes mitochondrion, complete genome*	15679	circular
NC_026560.1	*Austropotamobius pallipes*	*Austropotamobius pallipes mitochondrion, complete genome*	15679	circular
KR476914.1	*Ballerus sapa*	*Ballerus sapa isolate Balsap1 16S ribosomal RNA gene, partial sequence; mitochondrial*	510	linear

KR476833.1	*Ballerus sapa*	*Ballerus sapa isolate Balsap2 16S ribosomal RNA gene, partial sequence; mitochondrial*	516	linear
JN863593.1	*Barbodes wynaadensis*	*Barbus wynaadensis voucher NBFGR-CHN-NW2 16S ribosomal RNA gene, partial sequence; mitochondrial*	565	linear
AB238965.1	*Barbus barbus*	*Barbus barbus mitochondrial DNA, complete genome*	16600	circular
NC_008654.1	*Barbus barbus*	*Barbus barbus mitochondrion, complete genome*	16600	circular
KP712630.1	*Barbus ciscaucasicus*	*Barbus ciscaucasicus isolate CTOL00102 16S ribosomal RNA gene, partial sequence; mitochondrial*	591	linear
AP011322.1	*Barbus ciscaucasicus*	*Barbus ciscaucasicus mitochondrial DNA, complete genome, except for D-loop*	15664	linear
AJ247065.1	*Barbus fluviatilis*	*Barbus fluviatilis partial mitochondrial 16S rRNA gene*	432	linear
AJ247048.1	*Barbus meridionalis*	*Barbus meridionalis partial mitochondrial 16S rRNA gene, isolate 1*	424	linear
AJ247061.1	*Barbus meridionalis*	*Barbus meridionalis partial mitochondrial 16S rRNA gene, isolate 2*	425	linear
KR476836.1	*Blicca bjoerkna*	*Blicca bjoerkna isolate Blibjo1 16S ribosomal RNA gene, partial sequence; mitochondrial*	507	linear
KR476918.1	*Blicca bjoerkna*	*Blicca bjoerkna isolate Blibjo2 16S ribosomal RNA gene, partial sequence; mitochondrial*	508	linear
AP009304.1	*Blicca bjoerkna*	*Blicca bjoerkna mitochondrial DNA, complete genome*	16605	circular
AP011210.1	*Blicca bjoerkna*	*Blicca bjoerkna mitochondrial DNA, complete genome*	16603	circular
NC_020355.1	*Blicca bjoerkna*	*Blicca bjoerkna mitochondrion, complete genome*	16605	circular
AJ247064.1	*Blicca bjoerkna*	*Blicca bjoerkna partial mitochondrial 16S rRNA gene*	426	linear
KJ128679.1	*Blicca bjoerkna*	*Blicca bjoerkna voucher NRM:47767 16S ribosomal RNA gene, partial sequence; mitochondrial*	551	linear
KJ128680.1	*Blicca bjoerkna*	*Blicca bjoerkna voucher NRM:47967 16S ribosomal RNA gene, partial sequence; mitochondrial*	552	linear
AY500225.1	*Bombina pachypus*	*Bombina pachypus haplotype H1 16S ribosomal RNA gene, partial sequence; mitochondrial*	438	linear

AY500226.1	*Bombina pachypus*	*Bombina pachypus haplotype H2 16S ribosomal RNA gene, partial sequence; mitochondrial*	438	linear
AY500227.1	*Bombina pachypus*	*Bombina pachypus haplotype H3 16S ribosomal RNA gene, partial sequence; mitochondrial*	438	linear
AY500228.1	*Bombina pachypus*	*Bombina pachypus haplotype H4 16S ribosomal RNA gene, partial sequence; mitochondrial*	438	linear
EU531353.1	*Bombina pachypus*	*Bombina pachypus voucher TU-ST16 12S ribosomal RNA gene, partial sequence; tRNA-Val gene, complete sequence; and 16S ribosomal RNA gene, partial sequence; mitochondrial*	1956	linear
GQ380402.1	*Bufo bufo*	*Bufo bufo 16S ribosomal RNA gene, partial sequence; mitochondrial*	540	linear
MG199041.1	*Bufo bufo*	*Bufo bufo 16S ribosomal RNA gene, partial sequence; mitochondrial*	504	linear
AY555020.1	*Bufo bufo*	*Bufo bufo from Italy 16S ribosomal RNA gene, partial sequence; mitochondrial*	936	linear
JN647221.1	*Bufo spinosus*	*Bufo spinosus isolate BspinBB10 16S ribosomal RNA gene, partial sequence; mitochondrial*	513	linear
JN653292.1	*Bufo torrenticola*	*Bufo torrenticola isolate BtorrBB100 16S ribosomal RNA gene, partial sequence; mitochondrial*	505	linear
JQ348764.1	*Bufo verrucosissimus*	*Bufo verrucosissimus isolate 124 16S ribosomal RNA gene, partial sequence; mitochondrial*	565	linear
EU497418.1	*Bufotes balearicus*	*Bufo balearicus isolate B6 16S ribosomal RNA gene, partial sequence; mitochondrial*	610	linear
EU497419.1	*Bufotes balearicus*	*Bufo balearicus isolate B7 16S ribosomal RNA gene, partial sequence; mitochondrial*	610	linear
EU497420.1	*Bufotes balearicus*	*Bufo balearicus isolate B8 16S ribosomal RNA gene, partial sequence; mitochondrial*	610	linear
EU497421.1	*Bufotes balearicus*	*Bufo balearicus isolate B9 16S ribosomal RNA gene, partial sequence; mitochondrial*	610	linear
EU497422.1	*Bufotes balearicus*	*Bufo balearicus isolate B10 16S ribosomal RNA gene, partial sequence; mitochondrial*	610	linear

EU497423.1	*Bufotes balearicus*	*Bufo balearicus isolate B11 16S ribosomal RNA gene, partial sequence; mitochondrial*	609	linear
EU497424.1	*Bufotes balearicus*	*Bufo balearicus isolate B12 16S ribosomal RNA gene, partial sequence; mitochondrial*	609	linear
EU497425.1	*Bufotes balearicus*	*Bufo balearicus isolate B13 16S ribosomal RNA gene, partial sequence; mitochondrial*	609	linear
EU497426.1	*Bufotes balearicus*	*Bufo balearicus isolate B14 16S ribosomal RNA gene, partial sequence; mitochondrial*	609	linear
EU497427.1	*Bufotes balearicus*	*Bufo balearicus isolate B15 16S ribosomal RNA gene, partial sequence; mitochondrial*	609	linear
EU497428.1	*Bufotes balearicus*	*Bufo balearicus isolate B16 16S ribosomal RNA gene, partial sequence; mitochondrial*	609	linear
EU497429.1	*Bufotes balearicus*	*Bufo balearicus isolate B17 16S ribosomal RNA gene, partial sequence; mitochondrial*	609	linear
EU497430.1	*Bufotes balearicus*	*Bufo balearicus isolate B18 16S ribosomal RNA gene, partial sequence; mitochondrial*	610	linear
EU497431.1	*Bufotes balearicus*	*Bufo balearicus isolate B19 16S ribosomal RNA gene, partial sequence; mitochondrial*	610	linear
EU497432.1	*Bufotes balearicus*	*Bufo balearicus isolate B20 16S ribosomal RNA gene, partial sequence; mitochondrial*	609	linear
EU497433.1	*Bufotes balearicus*	*Bufo balearicus isolate B21 16S ribosomal RNA gene, partial sequence; mitochondrial*	610	linear
EU497434.1	*Bufotes balearicus*	*Bufo balearicus isolate B22 16S ribosomal RNA gene, partial sequence; mitochondrial*	610	linear
EU497435.1	*Bufotes balearicus*	*Bufo balearicus isolate B23 16S ribosomal RNA gene, partial sequence; mitochondrial*	610	linear
EU497436.1	*Bufotes balearicus*	*Bufo balearicus isolate B24 16S ribosomal RNA gene, partial sequence; mitochondrial*	610	linear
EU497437.1	*Bufotes balearicus*	*Bufo balearicus isolate B25 16S ribosomal RNA gene, partial sequence; mitochondrial*	610	linear
EU497462.1	*Bufotes balearicus*	*Bufo balearicus isolate B50 16S ribosomal RNA gene, partial sequence; mitochondrial*	609	linear
EU497463.1	*Bufotes balearicus*	*Bufo balearicus isolate B51 16S ribosomal RNA gene, partial sequence; mitochondrial*	609	linear

EU497464.1	*Bufotes balearicus*	*Bufo balearicus isolate B52 16S ribosomal RNA gene, partial sequence; mitochondrial*	609	linear
EU497465.1	*Bufotes balearicus*	*Bufo balearicus isolate B53 16S ribosomal RNA gene, partial sequence; mitochondrial*	609	linear
EU497466.1	*Bufotes balearicus*	*Bufo balearicus isolate B54 16S ribosomal RNA gene, partial sequence; mitochondrial*	609	linear
EU497467.1	*Bufotes balearicus*	*Bufo balearicus isolate B55 16S ribosomal RNA gene, partial sequence; mitochondrial*	609	linear
EU497468.1	*Bufotes balearicus*	*Bufo balearicus isolate B56 16S ribosomal RNA gene, partial sequence; mitochondrial*	609	linear
EU497469.1	*Bufotes balearicus*	*Bufo balearicus isolate B57 16S ribosomal RNA gene, partial sequence; mitochondrial*	609	linear
EU497470.1	*Bufotes balearicus*	*Bufo balearicus isolate B58 16S ribosomal RNA gene, partial sequence; mitochondrial*	609	linear
EU497471.1	*Bufotes balearicus*	*Bufo balearicus isolate B59 16S ribosomal RNA gene, partial sequence; mitochondrial*	609	linear
EU497472.1	*Bufotes balearicus*	*Bufo balearicus isolate B60 16S ribosomal RNA gene, partial sequence; mitochondrial*	609	linear
EU497473.1	*Bufotes balearicus*	*Bufo balearicus isolate B61 16S ribosomal RNA gene, partial sequence; mitochondrial*	609	linear
EU497474.1	*Bufotes balearicus*	*Bufo balearicus isolate B62 16S ribosomal RNA gene, partial sequence; mitochondrial*	609	linear
EU497475.1	*Bufotes balearicus*	*Bufo balearicus isolate B63 16S ribosomal RNA gene, partial sequence; mitochondrial*	609	linear
EU497476.1	*Bufotes balearicus*	*Bufo balearicus isolate B64 16S ribosomal RNA gene, partial sequence; mitochondrial*	609	linear
EU497477.1	*Bufotes balearicus*	*Bufo balearicus isolate B65 16S ribosomal RNA gene, partial sequence; mitochondrial*	609	linear
EU497478.1	*Bufotes balearicus*	*Bufo balearicus isolate B66 16S ribosomal RNA gene, partial sequence; mitochondrial*	609	linear
EU497479.1	*Bufotes balearicus*	*Bufo balearicus isolate B67 16S ribosomal RNA gene, partial sequence; mitochondrial*	609	linear
KC984245.1	*Carassius auratus*	*Carassius auratus 16S ribosomal RNA gene, partial sequence; mitochondrial*	229	linear

KU238073.1	*Carassius auratus*	*Carassius auratus 16S ribosomal RNA gene, partial sequence; mitochondrial*	612	linear
MH161806.1	*Carassius auratus*	*Carassius auratus 16S ribosomal RNA gene, partial sequence; mitochondrial*	495	linear
KJ476998.1	*Carassius auratus*	*Carassius auratus mitochondrion, complete genome*	16580	circular
KJ874428.1	*Carassius auratus*	*Carassius auratus mitochondrion, complete genome*	16580	circular
MF443758.1	*Carassius auratus auratus*	*Carassius auratus auratus isolate J1 mitochondrion, complete genome*	16580	circular
MF443759.1	*Carassius auratus auratus*	*Carassius auratus auratus isolate J2 mitochondrion, complete genome*	16580	circular
MF443760.1	*Carassius auratus auratus*	*Carassius auratus auratus isolate J3 mitochondrion, complete genome*	16580	circular
MF443761.1	*Carassius auratus auratus*	*Carassius auratus auratus isolate J4 mitochondrion, complete genome*	16580	circular
MF443762.1	*Carassius auratus auratus*	*Carassius auratus auratus isolate J5 mitochondrion, complete genome*	16580	circular
MF443763.1	*Carassius auratus auratus*	*Carassius auratus auratus isolate J6 mitochondrion, complete genome*	16580	circular
MF443764.1	*Carassius auratus auratus*	*Carassius auratus auratus isolate J7 mitochondrion, complete genome*	16580	circular
MF443765.1	*Carassius auratus auratus*	*Carassius auratus auratus isolate J8 mitochondrion, complete genome*	16580	circular
AB111951.1	*Carassius auratus auratus*	*Carassius auratus auratus mitochondrial DNA, complete genome*	16580	linear
AP011236.1	*Carassius auratus auratus*	*Carassius auratus auratus mitochondrial DNA, complete genome*	16580	circular
AB379915.1	*Carassius auratus auratus*	*Carassius auratus auratus mitochondrial DNA, partial sequence, isolate: G18*	9292	linear
AB379916.1	*Carassius auratus auratus*	*Carassius auratus auratus mitochondrial DNA, partial sequence, isolate: G19*	9294	linear
KR476838.1	*Carassius carassius*	*Carassius carassius isolate Carcar1 16S ribosomal RNA gene, partial sequence; mitochondrial*	512	linear

KR476920.1	*Carassius carassius*	*Carassius carassius isolate Carcar2 16S ribosomal RNA gene, partial sequence; mitochondrial*	216	linear
AY714387.1	*Carassius carassius*	*Carassius carassius mitochondrion, complete genome*	16580	circular
JQ911695.1	*Carassius carassius*	*Carassius carassius mitochondrion, complete genome*	16597	circular
KX781320.1	*Carassius carassius*	*Carassius carassius mitochondrion, complete genome*	16580	circular
KJ128723.1	*Carassius carassius*	*Carassius carassius voucher NRM:47877 16S ribosomal RNA gene, partial sequence; mitochondrial*	535	linear
KJ128724.1	*Carassius carassius*	*Carassius carassius voucher NRM:52860 16S ribosomal RNA gene, partial sequence; mitochondrial*	555	linear
KR476925.1	*Cobitis taenia*	*Cobitis taenia isolate Cobtae1 16S ribosomal RNA gene, partial sequence; mitochondrial*	512	linear
KR476844.1	*Cobitis taenia*	*Cobitis taenia isolate Cobtae2 16S ribosomal RNA gene, partial sequence; mitochondrial*	514	linear
AJ247080.1	*Cobitis taenia*	*Cobitis taenia partial mitochondrial 16S rRNA gene*	427	linear
KJ128742.1	*Cobitis taenia*	*Cobitis taenia voucher NRM:51845 16S ribosomal RNA gene, partial sequence; mitochondrial*	555	linear
KJ128743.1	*Cobitis taenia*	*Cobitis taenia voucher NRM:52492 16S ribosomal RNA gene, partial sequence; mitochondrial*	555	linear
AJ275218.1	*Corallotaenia intermedia*	*Corallotaenia intermedia mitochondrial partial 16S rRNA gene*	427	linear
DQ845935.1	*Cyprinus carpio*	*Cyprinus carpio 16S ribosomal RNA gene, partial sequence; mitochondrial*	1678	linear
DQ983941.1	*Cyprinus carpio*	*Cyprinus carpio 16S ribosomal RNA gene, partial sequence; mitochondrial*	612	linear
AP009047.1	*Cyprinus carpio*	*Cyprinus carpio mitochondrial DNA, complete genome*	16580	circular
AB741888.1	*Cyprinus carpio*	*Cyprinus carpio mitochondrial gene for 16S ribosomal RNA, partial sequence, country: Japan: Kochi, Shimanto*	623	linear
KF856965.1	*Cyprinus carpio*	*Cyprinus carpio mitochondrion, complete genome*	16581	circular

JN105352.1	*Cyprinus carpio carpio*	*Cyprinus carpio carpio mitochondrion, complete genome*	16581	circular
KJ168065.1	*Dicentrarchus labrax*	*Dicentrarchus labrax isolate DLmgm57 mitochondrion, complete genome*	18253	circular
NC_026074.1	*Dicentrarchus labrax*	*Dicentrarchus labrax isolate DLmgm57 mitochondrion, complete genome*	18253	circular
KJ168064.1	*Dicentrarchus labrax*	*Dicentrarchus labrax isolate DLP8F mitochondrion, complete genome*	17704	circular
FN687953.1	*Dicentrarchus labrax*	*Dicentrarchus labrax mitochondrial partial 16S rRNA gene, isolate 489*	489	linear
GQ485284.1	*Dicentrarchus labrax*	*Dicentrarchus labrax voucher 460 16S ribosomal RNA gene, partial sequence; mitochondrial*	554	linear
HQ731430.1	*Dicentrarchus labrax*	*Dicentrarchus labrax voucher GO905 16S ribosomal RNA gene, partial sequence; mitochondrial*	1315	linear
KJ128761.1	*Dicentrarchus labrax*	*Dicentrarchus labrax voucher NRM:49173 16S ribosomal RNA gene, partial sequence; mitochondrial*	554	linear
NC_031617.1	*Enteromius eburneensis*	*Barbus eburneensis mitochondrial DNA, complete genome*	16678	circular
AP011377.1	*Enteromius fasciolatus*	*Barbus fasciolatus mitochondrial DNA, complete genome*	16566	circular
NC_033914.1	*Enteromius pobeguini*	*Barbus pobeguini mitochondrial DNA, complete genome*	16933	circular
AB239600.1	*Enteromius trimaculatus*	*Barbus trimaculatus mitochondrial DNA, complete genome*	16417	circular
AF060446.1	*Esox lucius*	*Esox lucius 16S ribosomal RNA gene, mitochondrial gene for mitochondrial RNA, partial sequence*	565	linear
AF262309.1	*Esox lucius*	*Esox lucius isolate 1 16S ribosomal RNA gene, partial sequence; mitochondrial gene for mitochondrial product*	370	linear
KR476850.1	*Esox lucius*	*Esox lucius isolate Esoluc1 16S ribosomal RNA gene, partial sequence; mitochondrial*	453	linear
KR476932.1	*Esox lucius*	*Esox lucius isolate Esoluc3 16S ribosomal RNA gene, partial sequence; mitochondrial*	508	linear
AP004103.1	*Esox lucius*	*Esox lucius mitochondrial DNA, complete genome*	16695	circular
NC_004593.1	*Esox lucius*	*Esox lucius mitochondrion, complete genome*	16695	circular

FJ425099.1	*Esox lucius*	*Esox lucius voucher BBGY 101 16S ribosomal RNA gene, partial sequence; mitochondrial*	519	linear
KJ128767.1	*Esox lucius*	*Esox lucius voucher NRM:51663 16S ribosomal RNA gene, partial sequence; mitochondrial*	553	linear
U80050.1	*Gambusia holbrooki*	*Gambusia holbrooki 16S ribosomal RNA gene, mitochondrial gene encoding mitochondrial rRNA, partial sequence*	448	linear
KM435020.1	*Gambusia holbrooki*	*Gambusia holbrooki 16S ribosomal RNA gene, partial sequence; mitochondrial*	316	linear
LC198798.1	*Gambusia holbrooki*	*Gambusia holbrooki mitochondrial gene for 16S ribosomal RNA, partial sequence, isolation_source: aquarium water sample 1*	81	linear
LC198799.1	*Gambusia holbrooki*	*Gambusia holbrooki mitochondrial gene for 16S ribosomal RNA, partial sequence, isolation_source: aquarium water sample 5*	81	linear
LC198797.1	*Gambusia holbrooki*	*Gambusia holbrooki mitochondrial gene for 16S ribosomal RNA, partial sequence, isolation_source: laboratory tissue sample G_holbrooki_010*	78	linear
LC198800.1	*Gambusia holbrooki*	*Gambusia holbrooki mitochondrial gene for 16S ribosomal RNA, partial sequence, isolation_source: water sample 1 from Ebro River point 1*	85	linear
LC198801.1	*Gambusia holbrooki*	*Gambusia holbrooki mitochondrial gene for 16S ribosomal RNA, partial sequence, isolation_source: water sample 2 from Ebro River point 2*	76	linear
LC198802.1	*Gambusia holbrooki*	*Gambusia holbrooki mitochondrial gene for 16S ribosomal RNA, partial sequence, isolation_source: water sample 3 from Ebro River point 3*	76	linear
LC198803.1	*Gambusia holbrooki*	*Gambusia holbrooki mitochondrial gene for 16S ribosomal RNA, partial sequence, isolation_source: water sample 4 from Ebro River point 4*	77	linear
LC198804.1	*Gambusia holbrooki*	*Gambusia holbrooki mitochondrial gene for 16S ribosomal RNA, partial sequence, isolation_source:*	77	linear

		water sample 5 from Ebro River point 5		
KP013085.1	*Gambusia holbrooki*	*Gambusia holbrooki voucher LodgeLab Gholbrooki_1 mitochondrion, complete genome*	16611	circular
NC_028274.1	*Gambusia holbrooki*	*Gambusia holbrooki voucher LodgeLab Gholbrooki_1 mitochondrion, complete genome*	16611	circular
KP013115.1	*Gambusia holbrooki*	*Gambusia holbrooki voucher LodgeLab Gholbrooki_2 mitochondrion, complete genome*	16611	circular
DQ027919.1	*Gasterosteus aculeatus*	*Gasterosteus aculeatus 16S ribosomal RNA gene, partial sequence; mitochondrial*	540	linear
AF355030.1	*Gasterosteus aculeatus*	*Gasterosteus aculeatus 16S ribosomal RNA gene, partial sequence; mitochondrial gene for mitochondrial product*	523	linear
AY283358.1	*Gasterosteus aculeatus*	*Gasterosteus aculeatus haplotype Ga1 16S ribosomal RNA gene, partial sequence; mitochondrial gene for mitochondrial product*	418	linear
KJ627974.1	*Gasterosteus aculeatus*	*Gasterosteus aculeatus haplotype Gac1 16S ribosomal RNA gene, partial sequence; mitochondrial*	600	linear
DQ077967.1	*Gasterosteus aculeatus*	*Gasterosteus aculeatus isolate A9 16S ribosomal RNA gene, partial sequence; mitochondrial*	544	linear
DQ077966.1	*Gasterosteus aculeatus*	*Gasterosteus aculeatus isolate B1 16S ribosomal RNA gene, partial sequence; mitochondrial*	590	linear
DQ077965.1	*Gasterosteus aculeatus*	*Gasterosteus aculeatus isolate B2 16S ribosomal RNA gene, partial sequence; mitochondrial*	587	linear
DQ077964.1	*Gasterosteus aculeatus*	*Gasterosteus aculeatus isolate B3 16S ribosomal RNA gene, partial sequence; mitochondrial*	566	linear
DQ077963.1	*Gasterosteus aculeatus*	*Gasterosteus aculeatus isolate B4 16S ribosomal RNA gene, partial sequence; mitochondrial*	590	linear
DQ077962.1	*Gasterosteus aculeatus*	*Gasterosteus aculeatus isolate B5 16S ribosomal RNA gene, partial sequence; mitochondrial*	586	linear
MN122917.1	*Gasterosteus aculeatus*	*Gasterosteus aculeatus isolate DM180 mitochondrion, partial genome*	16599	linear
MH205729.1	*Gasterosteus aculeatus*	*Gasterosteus aculeatus mitochondrion, complete genome*	16543	circular
EF458412.1	*Gasterosteus aculeatus*	*Gasterosteus aculeatus voucher UW:049025 16S ribosomal RNA*	580	linear

		gene, partial sequence; mitochondrial		
AB070241.1	*Hemibarbus barbus*	*Hemibarbus barbus mitochondrial DNA, complete genome*	16681	circular
KF991279.1	*Humerana lateralis*	*Pelophylax lateralis voucher ZFMK:92555 16S ribosomal RNA gene, partial sequence; mitochondrial*	550	linear
KM282485.1	*Lepomis gibbosus*	*Lepomis gibbosus 16S ribosomal RNA gene, partial sequence; mitochondrial*	338	linear
AY742524.1	*Lepomis gibbosus*	*Lepomis gibbosus isolate LgibA 16S ribosomal RNA gene, partial sequence; mitochondrial*	1682	linear
NC_028284.1	*Lepomis gibbosus*	*Lepomis gibbosus voucher LodgeLab Lgibbosus_1 mitochondrion, complete genome*	16503	circular
MK013969.1	*Lissotriton italicus*	*Lissotriton italicus 16S ribosomal RNA gene, partial sequence; mitochondrial*	503	linear
DQ092269.1	*Lissotriton italicus*	*Triturus italicus isolate E1806.18 16S ribosomal RNA gene, partial sequence; mitochondrial*	557	linear
MH136825.1	*Luciobarbus capito*	*Luciobarbus capito isolate DLB-B mitochondrion, complete genome*	16603	circular
MH136824.1	*Luciobarbus capito*	*Luciobarbus capito isolate DLB-O mitochondrion, complete genome*	16607	circular
JX987313.1	*Luciobarbus capito*	*Luciobarbus capito mitochondrion, complete genome*	16603	circular
JX987313.1	*Luciobarbus capito*	*Luciobarbus capito mitochondrion, complete genome*	16603	circular
NC_020338.1	*Luciobarbus capito*	*Luciobarbus capito mitochondrion, complete genome*	16603	circular
KX348041.1	*Luciobarbus rifensis*	*Luciobarbus rifensis voucher MNCN-235026 mitochondrion, complete genome*	16607	circular
AP011196.1	*Luciobarbus sclateri*	*Luciobarbus sclateri mitochondrial DNA, complete genome*	16608	circular
NC_031532.1	*Luciobarbus sclateri*	*Luciobarbus sclateri mitochondrial DNA, complete genome*	16608	circular
AF042466.1	*Micropterus salmoides*	*Micropterus salmoides 16S ribosomal RNA gene, mitochondrial gene for mitochondrial RNA, partial sequence*	401	linear
KM282489.1	*Micropterus salmoides*	*Micropterus salmoides 16S ribosomal RNA gene, partial sequence; mitochondrial*	339	linear
AP014537.1	*Micropterus salmoides*	*Micropterus salmoides mitochondrial DNA, almost complete genome*	16165	circular

NC_008106.1	*Micropterus salmoides*	*Micropterus salmoides mitochondrion, complete genome*	16484	circular
DQ307686.1	*Mugil cephalus*	*Mugil cephalus 16S ribosomal RNA gene, partial sequence; mitochondrial*	1317	linear
EU239812.1	*Mugil cephalus*	*Mugil cephalus 16S ribosomal RNA gene, partial sequence; mitochondrial*	610	linear
JQ060816.2	*Mugil cephalus*	*Mugil cephalus isolate 319 16S ribosomal RNA gene, partial sequence; mitochondrial*	1339	linear
KM406308.1	*Neogobius nigricans*	*Neogobius nigricans voucher Pn04 16S ribosomal RNA gene, partial sequence; mitochondrial*	426	linear
AF067270.1	*Neogobius nigricans*	*Padogobius nigricans 16S ribosomal RNA gene, mitochondrial gene for mitochondrial RNA, partial sequence*	491	linear
NC_001717.1	*Oncorhynchus mykiss*	*Oncorhynchus mykiss mitochondrion complete genome*	16642	circular
L29771.1	*Oncorhynchus mykiss*	*Oncorhynchus mykiss mitochondrion complete genome*	16642	circular
KM406309.1	*Padogobius bonelli*	*Padogobius bonelli voucher Pn09 16S ribosomal RNA gene, partial sequence; mitochondrial*	423	linear
AF067274.1	*Padogobius martensii*	*Padogobius martensi 16S ribosomal RNA gene, mitochondrial gene for mitochondrial RNA, partial sequence*	483	linear
EF218652.1	*Padogobius martensii*	*Padogobius martensii 16S ribosomal RNA gene, partial sequence; mitochondrial*	545	linear
NC_029200.1	*Pelophylax bedriagae*	*Pelophylax bedriagae isolate GM-J0142 mitochondrion, complete genome*	17968	circular
NC_029196.1	*Pelophylax cf. bedriagae AFL1*	*Pelophylax cf. bedriagae AFL1 mitochondrion, complete genome*	17977	circular
NC_029199.1	*Pelophylax cf. terentievi MPFC1736*	*Pelophylax cf. terentievi MPFC1736 mitochondrion, complete genome*	17990	circular
NC_025575.1	*Pelophylax cretensis*	*Pelophylax cretensis isolate CR03 mitochondrion, complete genome*	17829	circular
NC_026894.1	*Pelophylax epeiroticus*	*Pelophylax epeiroticus isolate MPFC1392 mitochondrion, complete genome*	18030	circular
MN122883.1	*Pelophylax esculentus*	*Pelophylax esculentus mitochondrion, partial genome*	16525	linear

NC_026895.1	*Pelophylax kurtmuelleri*	*Pelophylax kurtmuelleri isolate MPFC1475 mitochondrion, complete genome*	18020	circular
MH410480.1	*Pelophylax lessonae*	*Pelophylax lessonae voucher F59 16S ribosomal RNA gene, partial sequence; mitochondrial*	538	linear
KX269216.1	*Pelophylax nigromaculatus*	*Pelophylax nigromaculatus isolate SCUM045199CJ 12S ribosomal RNA gene, partial sequence; tRNA-Val gene, complete sequence; and 16S ribosomal RNA gene, partial sequence; mitochondrial*	1965	linear
KY762042.1	*Pelophylax perezi*	*Pelophylax perezi voucher SPM2256 16S ribosomal RNA gene, partial sequence; mitochondrial*	538	linear
JQ815307.1	*Pelophylax plancyi*	*Pelophylax plancyi voucher NIBRAM0000000038 16S ribosomal RNA gene, partial sequence; mitochondrial*	558	linear
LC389209.1	*Pelophylax porosus brevipodus*	*Pelophylax porosus brevipodus Pp1 mitochondrial gene for 16S rRNA, partial sequence*	537	linear
MH410472.1	*Pelophylax ridibundus*	*Pelophylax ridibundus voucher F51 16S ribosomal RNA gene, partial sequence; mitochondrial*	538	linear
KP177567.1	*Pelophylax saharicus*	*Pelophylax saharicus voucher MNHN:2011-558 16S ribosomal RNA gene, partial sequence; mitochondrial*	459	linear
U87422.1	*Perca fluviatilis*	*Perca fluviatilis 16S mitochondrial ribosomal RNA mitochondrial gene, partial sequence*	430	linear
JQ999985.1	*Perca fluviatilis*	*Perca fluviatilis 16S ribosomal RNA gene, partial sequence; mitochondrial*	1103	linear
AF518221.1	*Perca fluviatilis*	*Perca fluviatilis 16S ribosomal RNA gene, partial sequence; mitochondrial gene for mitochondrial product*	784	linear
AY141442.1	*Perca fluviatilis*	*Perca fluviatilis 16S ribosomal RNA gene, partial sequence; mitochondrial gene for mitochondrial product*	388	linear
AY254567.1	*Perca fluviatilis*	*Perca fluviatilis 16S ribosomal RNA gene, partial sequence; mitochondrial gene for mitochondrial product*	507	linear

MH271189.1	*Perca fluviatilis*	*Perca fluviatilis haplotype 01-SK03 16S ribosomal RNA gene, partial sequence; mitochondrial*	541	linear
MH271188.1	*Perca fluviatilis*	*Perca fluviatilis haplotype 05-IT02 16S ribosomal RNA gene, partial sequence; mitochondrial*	541	linear
MH271187.1	*Perca fluviatilis*	*Perca fluviatilis haplotype 09-CZ05 16S ribosomal RNA gene, partial sequence; mitochondrial*	541	linear
MG969725.1	*Perca fluviatilis*	*Perca fluviatilis haplotype H1 16S ribosomal RNA gene, partial sequence; mitochondrial*	541	linear
MG969726.1	*Perca fluviatilis*	*Perca fluviatilis haplotype H2 16S ribosomal RNA gene, partial sequence; mitochondrial*	540	linear
MG969727.1	*Perca fluviatilis*	*Perca fluviatilis haplotype H3 16S ribosomal RNA gene, partial sequence; mitochondrial*	541	linear
MG969728.1	*Perca fluviatilis*	*Perca fluviatilis haplotype H4 16S ribosomal RNA gene, partial sequence; mitochondrial*	540	linear
MG969731.1	*Perca fluviatilis*	*Perca fluviatilis haplotype H5 16S ribosomal RNA gene, partial sequence; mitochondrial*	541	linear
MG969732.1	*Perca fluviatilis*	*Perca fluviatilis haplotype H6 16S ribosomal RNA gene, partial sequence; mitochondrial*	541	linear
MG969733.1	*Perca fluviatilis*	*Perca fluviatilis haplotype H7 16S ribosomal RNA gene, partial sequence; mitochondrial*	541	linear
MG969734.1	*Perca fluviatilis*	*Perca fluviatilis haplotype H8 16S ribosomal RNA gene, partial sequence; mitochondrial*	541	linear
MG969735.1	*Perca fluviatilis*	*Perca fluviatilis haplotype H9 16S ribosomal RNA gene, partial sequence; mitochondrial*	541	linear
MG969736.1	*Perca fluviatilis*	*Perca fluviatilis haplotype H10 16S ribosomal RNA gene, partial sequence; mitochondrial*	541	linear
MG969737.1	*Perca fluviatilis*	*Perca fluviatilis haplotype H11 16S ribosomal RNA gene, partial sequence; mitochondrial*	541	linear
KR476954.1	*Perca fluviatilis*	*Perca fluviatilis isolate Perflu1 16S ribosomal RNA gene, partial sequence; mitochondrial*	510	linear
KR476873.1	*Perca fluviatilis*	*Perca fluviatilis isolate Perflu9 16S ribosomal RNA gene, partial sequence; mitochondrial*	511	linear
AP005995.1	*Perca fluviatilis*	*Perca fluviatilis mitochondrial DNA, complete genome*	16537	circular

KM410088.1	*Perca fluviatilis*	*Perca fluviatilis mitochondrion, complete genome*	16537	circular
NC_026313.1	*Perca fluviatilis*	*Perca fluviatilis mitochondrion, complete genome*	16537	circular
HQ731432.1	*Perca fluviatilis*	*Perca fluviatilis voucher KU2567 16S ribosomal RNA gene, partial sequence; mitochondrial*	568	linear
MG969729.1	*Perca schrenkii*	*Perca schrenkii haplotype H1 16S ribosomal RNA gene, partial sequence; mitochondrial*	541	linear
MG969730.1	*Perca schrenkii*	*Perca schrenkii haplotype H2 16S ribosomal RNA gene, partial sequence; mitochondrial*	541	linear
HQ623651.1	*Petromyzon marinus*	*Petromyzon marinus 16S ribosomal RNA gene, partial sequence; mitochondrial*	90	linear
EU404074.1	*Petromyzon marinus*	*Petromyzon marinus haplotype Pma71 16S ribosomal RNA gene, partial sequence; mitochondrial*	463	linear
U11880.1	*Petromyzon marinus*	*Petromyzon marinus mitochondrion, complete genome*	16201	circular
KJ128854.1	*Petromyzon marinus*	*Petromyzon marinus voucher NRM:50292 16S ribosomal RNA gene, partial sequence; mitochondrial*	525	linear
KJ533240.1	*Plotosus lineatus*	*Plotosus lineatus isolate QP1 16S ribosomal RNA gene, partial sequence; mitochondrial*	563	linear
KJ533241.1	*Plotosus lineatus*	*Plotosus lineatus isolate QP2 16S ribosomal RNA gene, partial sequence; mitochondrial*	563	linear
KU213641.1	*Plotosus lineatus*	*Plotosus lineatus mitochondrion, complete genome*	16480	circular
NC_029714.1	*Plotosus lineatus*	*Plotosus lineatus mitochondrion, complete genome*	16480	circular
DQ532941.1	*Pomatomus saltatrix*	*Pomatomus saltatrix 16S large subunit ribosomal RNA gene, partial sequence; mitochondrial*	549	linear
FJ374805.1	*Pomatomus saltatrix*	*Pomatomus saltatrix 16S ribosomal RNA gene, partial sequence; mitochondrial*	1613	linear
HQ623631.1	*Pomatomus saltatrix*	*Pomatomus saltatrix 16S ribosomal RNA gene, partial sequence; mitochondrial*	119	linear
AF055612.1	*Pomatomus saltatrix*	*Pomatomus saltatrix 16S small subunit ribosomal RNA gene, partial sequence, mitochondrial gene for mitochondrial RNA*	571	linear
AB355904.1	*Pomatomus saltatrix*	*Pomatomus saltatrix mitochondrial DNA, complete genome*	16544	circular

NC_022507.1	*Pomatomus saltatrix*	*Pomatomus saltatrix mitochondrion, complete genome*	16544	circular
JQ938975.1	*Pomatomus saltatrix*	*Pomatomus saltatrix voucher KU3992 16S ribosomal RNA gene, partial sequence; mitochondrial*	679	linear
EU410419.1	*Pomatomus saltatrix*	*Pomatomus saltatrix voucher Pomsal/58 16S ribosomal RNA gene, partial sequence; mitochondrial*	556	linear
DQ447706.1	*Protochondrostoma genei*	*Chondrostoma genei 16S ribosomal RNA gene, partial sequence; mitochondrial*	566	linear
EU853289.1	*Protochondrostoma genei*	*Chondrostoma genei isolate ProgenBO1 16S ribosomal RNA gene, partial sequence; mitochondrial*	540	linear
EU853290.1	*Protochondrostoma genei*	*Chondrostoma genei isolate ProgenBO2 16S ribosomal RNA gene, partial sequence; mitochondrial*	540	linear
AJ247057.1	*Protochondrostoma genei*	*Chondrostoma genei partial mitochondrial 16S rRNA gene*	425	linear
DQ447703.1	*Pseudochondrostoma polylepis*	*Chondrostoma polylepis 16S ribosomal RNA gene, partial sequence; mitochondrial*	581	linear
AP011282.1	*Pseudochondrostoma polylepis*	*Pseudochondrostoma polylepis mitochondrial DNA, complete genome*	16606	circular
NC_031574.1	*Pseudochondrostoma polylepis*	*Pseudochondrostoma polylepis mitochondrial DNA, complete genome*	16606	circular
GQ406307.1	*Pseudorasbora parva*	*Pseudorasbora parva 16S ribosomal RNA gene, partial sequence*	1682	linear
KU238078.1	*Pseudorasbora parva*	*Pseudorasbora parva 16S ribosomal RNA gene, partial sequence; mitochondrial*	619	linear
KJ135626.1	*Pseudorasbora parva*	*Pseudorasbora parva mitochondrion, complete genome*	16601	circular
NC_015614.1	*Pseudorasbora parva*	*Pseudorasbora parva mitochondrion, complete genome*	16600	circular
AB719223.1	*Pulchrana glandulosa*	*Rana glandulosa mitochondrial gene for 16S rRNA, partial sequence, specimen_voucher: KUHE:53618*	1170	linear
AY147942.1	*Rana graeca*	*Rana graeca 16S ribosomal RNA gene, partial sequence; mitochondrial gene for mitochondrial product*	1168	linear
AY043078.1	*Rana italica*	*Rana italica 16S ribosomal RNA gene, partial sequence;*	206	linear

		mitochondrial gene for mitochondrial product		
AY147945.1	*Rana italica*	*Rana italica 16S ribosomal RNA gene, partial sequence; mitochondrial gene for mitochondrial product*	1165	linear
KP222281.1	*Rana sylvatica*	*Rana sylvatica mitochondrion, complete genome*	17343	circular
MH536744.1	*Rana temporaria*	*Rana temporaria mitochondrion, complete genome*	16061	circular
AB430342.1	*Rugosa rugosa*	*Rana rugosa mitochondrial genes for 12S rRNA, tRNA-Val, 16S rRNA, partial and complete sequence, haplotype: XY*	2325	linear
EU853296.1	*Rutilus aula*	*Rutilus aula isolate Rutery 16S ribosomal RNA gene, partial sequence; mitochondrial*	539	linear
KR476883.1	*Rutilus meidingeri*	*Rutilus meidingeri isolate Rutmei1 16S ribosomal RNA gene, partial sequence; mitochondrial*	510	linear
KR476965.1	*Rutilus meidingeri*	*Rutilus meidingeri isolate Rutmei2 16S ribosomal RNA gene, partial sequence; mitochondrial*	512	linear
GQ406271.1	*Rutilus rutilus*	*Rutilus rutilus 16S ribosomal RNA gene, partial sequence*	1689	linear
AP010775.1	*Rutilus rutilus*	*Rutilus rutilus mitochondrial DNA, complete genome*	16606	circular
FJ465725.1	*Salaria fluviatilis*	*Salaria fluviatilis 16S ribosomal RNA gene, partial sequence; mitochondrial*	586	linear
AY098843.1	*Salaria fluviatilis*	*Salaria fluviatilis from Portugal 16S ribosomal RNA gene, partial sequence; mitochondrial gene for mitochondrial product*	574	linear
HQ623636.1	*Salmo trutta*	*Salmo trutta 16S ribosomal RNA gene, partial sequence; mitochondrial*	121	linear
KC441986.1	*Salmo trutta*	*Salmo trutta 16S ribosomal RNA gene, partial sequence; mitochondrial*	392	linear
LT617632.1	*Salmo trutta*	*Salmo trutta mitochondrial genome, specimen voucher MNHN_IC_1977_272_A*	16677	circular
LT617631.1	*Salmo trutta*	*Salmo trutta mitochondrial genome, specimen voucher MNHN_IC_A_7585*	16677	circular
JQ390057.1	*Salmo trutta*	*Salmo trutta mitochondrion, complete genome*	16677	circular
AF446373.1	*Salmo trutta fario*	*Salmo trutta fario isolate 2 pop-variant Dol-ME 16S ribosomal RNA gene, partial sequence;*	372	linear

		mitochondrial gene for mitochondrial product		
LC137015.1	*Salmo trutta fario*	*Salmo trutta fario mitochondrial DNA, complete genome, collection_date: 2014-11-19*	16687	circular
KT633607.1	*Salmo trutta fario*	*Salmo trutta fario mitochondrion, complete genome*	16677	circular
X77562.1	*Salmo trutta macrostigma*	*Salmo trutta macrostigma mitochondrial gene for 16S ribosomal RNA*	343	linear
X77521.1	*Salmo trutta macrostigma*	*Salmo trutta macrostigma mitochondrial gene for cytochrome b*	249	linear
AM910409.1	*Salmo trutta trutta*	*Salmo trutta trutta complete mitochondrial genome, specimen voucher Nor 00*	16677	circular
NC_010007.1	*Salmo trutta trutta*	*Salmo trutta trutta mitochondrion, complete genome*	16677	circular
EU853297.1	*Sarmarutilus rubilio*	*Rutilus rubilio isolate RutrubBO 16S ribosomal RNA gene, partial sequence; mitochondrial*	539	linear
EU853299.1	*Sarmarutilus rubilio*	*Rutilus rubilio isolate RutrubCH 16S ribosomal RNA gene, partial sequence; mitochondrial*	539	linear
EU853298.1	*Sarmarutilus rubilio*	*Rutilus rubilio isolate RutrubPG 16S ribosomal RNA gene, partial sequence; mitochondrial*	539	linear
AJ247059.1	*Sarmarutilus rubilio*	*Rutilus rubilio partial mitochondrial 16S rRNA gene*	425	linear
AJ247066.1	*Scardinius erythrophthalmus*	*Scardinius erythrophtalmus partial mitochondrial 16S rRNA gene, isolate 2*	428	linear
AF215479.1	*Scardinius erythrophthalmus*	*Scardinius erythrophthalmus 16S ribosomal RNA gene, partial sequence; mitochondrial*	572	linear
EF372635.1	*Scardinius erythrophthalmus*	*Scardinius erythrophthalmus 16S ribosomal RNA gene, partial sequence; mitochondrial*	484	linear
KR476897.1	*Scardinius erythrophthalmus*	*Scardinius erythrophthalmus isolate Scaery2 16S ribosomal RNA gene, partial sequence; mitochondrial*	517	linear
EU853295.1	*Scardinius erythrophthalmus*	*Scardinius erythrophthalmus isolate Scaery 16S ribosomal RNA gene, partial sequence; mitochondrial*	539	linear
AP011263.1	*Scardinius erythrophthalmus*	*Scardinius erythrophthalmus mitochondrial DNA, complete genome*	16607	circular

NC_031561.1	*Scardinius erythrophthalmus*	*Scardinius erythrophthalmus mitochondrial DNA, complete genome*	16607	circular
KJ128896.1	*Scardinius erythrophthalmus*	*Scardinius erythrophthalmus voucher NRM:48318 16S ribosomal RNA gene, partial sequence; mitochondrial*	553	linear
KJ128895.1	*Scardinius erythrophthalmus*	*Scardinius erythrophthalmus voucher NRM:48655 16S ribosomal RNA gene, partial sequence; mitochondrial*	553	linear
EF095593.1	*Sparus aurata*	*Sparus aurata 16S ribosomal RNA gene, partial sequence; mitochondrial*	391	linear
KC984230.1	*Sparus aurata*	*Sparus aurata 16S ribosomal RNA gene, partial sequence; mitochondrial*	229	linear
AF247432.1	*Sparus aurata*	*Sparus aurata 16S ribosomal RNA gene, partial sequence; mitochondrial gene for mitochondrial product*	576	linear
LK022698.1	*Sparus aurata*	*Sparus aurata mitochondrial complete genome*	16652	circular
NC_024236.1	*Sparus aurata*	*Sparus aurata mitochondrial complete genome*	16652	circular
FN688245.1	*Sparus aurata*	*Sparus aurata mitochondrial partial 16S rRNA gene, isolate 456*	489	linear
FN688246.1	*Sparus aurata*	*Sparus aurata mitochondrial partial 16S rRNA gene, isolate 459*	489	linear
FN688247.1	*Sparus aurata*	*Sparus aurata mitochondrial partial 16S rRNA gene, isolate 464*	489	linear
FN688248.1	*Sparus aurata*	*Sparus aurata mitochondrial partial 16S rRNA gene, isolate 789*	489	linear
FN688249.1	*Sparus aurata*	*Sparus aurata mitochondrial partial 16S rRNA gene, isolate 834*	489	linear
FN688243.1	*Sparus aurata*	*Sparus aurata mitochondrial partial 16S rRNA gene, isolate 1960*	489	linear
FN688244.1	*Sparus aurata*	*Sparus aurata mitochondrial partial 16S rRNA gene, isolate 1962*	489	linear
KT805959.1	*Sparus aurata*	*Sparus aurata mitochondrion, complete genome*	16652	circular
AJ247279.1	*Sparus aurata*	*Sparus aurata partial mitochondrial 16S rRNA gene*	479	linear
GQ485281.1	*Sparus aurata*	*Sparus aurata voucher 864 16S ribosomal RNA gene, partial sequence; mitochondrial*	552	linear
AJ247054.1	*Squalius cephalus*	*Leuciscus cephalus partial mitochondrial 16S rRNA gene*	864	linear

EU853292.1	*Squalius cephalus*	*Squalius cephalus isolate LeucepBO2 16S ribosomal RNA gene, partial sequence; mitochondrial*	539	linear
AP011214.1	*Squalius cephalus*	*Squalius cephalus mitochondrial DNA, complete genome*	16612	circular
NC_031540.1	*Squalius cephalus*	*Squalius cephalus mitochondrial DNA, complete genome*	16612	circular
EU530554.1	*Syngnathus abaster*	*Syngnathus abaster isolate c1 16S ribosomal RNA gene, partial sequence; mitochondrial*	519	linear
EU530555.1	*Syngnathus abaster*	*Syngnathus abaster isolate c2 16S ribosomal RNA gene, partial sequence; mitochondrial*	519	linear
EU530556.1	*Syngnathus abaster*	*Syngnathus abaster isolate c3 16S ribosomal RNA gene, partial sequence; mitochondrial*	519	linear
EU530557.1	*Syngnathus abaster*	*Syngnathus abaster isolate c4 16S ribosomal RNA gene, partial sequence; mitochondrial*	519	linear
JF306314.1	*Syngnathus abaster*	*Syngnathus abaster isolate cd01 16S ribosomal RNA gene, partial sequence; mitochondrial*	519	linear
JF306315.1	*Syngnathus abaster*	*Syngnathus abaster isolate cd02 16S ribosomal RNA gene, partial sequence; mitochondrial*	519	linear
JF306316.1	*Syngnathus abaster*	*Syngnathus abaster isolate cd03 16S ribosomal RNA gene, partial sequence; mitochondrial*	519	linear
JF306317.1	*Syngnathus abaster*	*Syngnathus abaster isolate cd04 16S ribosomal RNA gene, partial sequence; mitochondrial*	519	linear
JF306318.1	*Syngnathus abaster*	*Syngnathus abaster isolate cd05 16S ribosomal RNA gene, partial sequence; mitochondrial*	519	linear
JF306319.1	*Syngnathus abaster*	*Syngnathus abaster isolate cd06 16S ribosomal RNA gene, partial sequence; mitochondrial*	519	linear
JF306320.1	*Syngnathus abaster*	*Syngnathus abaster isolate cd07 16S ribosomal RNA gene, partial sequence; mitochondrial*	519	linear
JF306321.1	*Syngnathus abaster*	*Syngnathus abaster isolate cd08 16S ribosomal RNA gene, partial sequence; mitochondrial*	519	linear
JF306322.1	*Syngnathus abaster*	*Syngnathus abaster isolate cd09 16S ribosomal RNA gene, partial sequence; mitochondrial*	519	linear
JF306323.1	*Syngnathus abaster*	*Syngnathus abaster isolate cd10 16S ribosomal RNA gene, partial sequence; mitochondrial*	519	linear

JF306363.1	*Syngnathus abaster*	*Syngnathus abaster isolate em01 16S ribosomal RNA gene, partial sequence; mitochondrial*	519	linear
JF306364.1	*Syngnathus abaster*	*Syngnathus abaster isolate em02 16S ribosomal RNA gene, partial sequence; mitochondrial*	519	linear
JF306365.1	*Syngnathus abaster*	*Syngnathus abaster isolate em03 16S ribosomal RNA gene, partial sequence; mitochondrial*	519	linear
JF306366.1	*Syngnathus abaster*	*Syngnathus abaster isolate em04 16S ribosomal RNA gene, partial sequence; mitochondrial*	519	linear
JF306367.1	*Syngnathus abaster*	*Syngnathus abaster isolate em05 16S ribosomal RNA gene, partial sequence; mitochondrial*	519	linear
JF306368.1	*Syngnathus abaster*	*Syngnathus abaster isolate em06 16S ribosomal RNA gene, partial sequence; mitochondrial*	519	linear
JF306325.1	*Syngnathus abaster*	*Syngnathus abaster isolate sc02 16S ribosomal RNA gene, partial sequence; mitochondrial*	519	linear
JF306374.1	*Syngnathus abaster*	*Syngnathus abaster isolate ts01 16S ribosomal RNA gene, partial sequence; mitochondrial*	519	linear
JF306378.1	*Syngnathus abaster*	*Syngnathus abaster isolate ts04 16S ribosomal RNA gene, partial sequence; mitochondrial*	519	linear
JF306390.1	*Syngnathus abaster*	*Syngnathus abaster isolate vn05 16S ribosomal RNA gene, partial sequence; mitochondrial*	519	linear
JF306394.1	*Syngnathus abaster*	*Syngnathus abaster isolate vn09 16S ribosomal RNA gene, partial sequence; mitochondrial*	519	linear
JF306400.1	*Syngnathus abaster*	*Syngnathus abaster isolate vs05 16S ribosomal RNA gene, partial sequence; mitochondrial*	519	linear
AF355010.1	*Syngnathus abaster*	*Syngnathus abaster S23 16S ribosomal RNA gene, partial sequence; mitochondrial gene for mitochondrial product*	549	linear
DQ447688.1	*Telestes souffia*	*Leuciscus souffia 16S ribosomal RNA gene, partial sequence; mitochondrial*	557	linear
AJ247049.1	*Telestes souffia*	*Leuciscus soufia partial mitochondrial 16S rRNA gene, isolate 2*	423	linear
AJ247050.1	*Telestes souffia*	*Leuciscus soufia partial mitochondrial 16S rRNA gene, isolate 3*	423	linear

AJ247051.1	*Telestes souffia*	*Leuciscus soufia partial mitochondrial 16S rRNA gene, isolate 4*	423	linear
GQ406280.1	*Tinca tinca*	*Tinca tinca 16S ribosomal RNA gene, partial sequence*	1688	linear
KR476983.1	*Tinca tinca*	*Tinca tinca isolate Tintin1 16S ribosomal RNA gene, partial sequence; mitochondrial*	512	linear
KR476902.1	*Tinca tinca*	*Tinca tinca isolate Tintin2 16S ribosomal RNA gene, partial sequence; mitochondrial*	517	linear
EU853302.1	*Tinca tinca*	*Tinca tinca isolate Tintin 16S ribosomal RNA gene, partial sequence; mitochondrial*	538	linear
AB218686.1	*Tinca tinca*	*Tinca tinca mitochondrial DNA, complete genome*	16612	circular
NC_008648.1	*Tinca tinca*	*Tinca tinca mitochondrion, complete genome*	16612	circular
AJ247053.1	*Tinca tinca*	*Tinca tinca partial mitochondrial 16S rRNA gene*	423	linear
KJ128924.1	*Tinca tinca*	*Tinca tinca voucher NRM:52453 16S ribosomal RNA gene, partial sequence; mitochondrial*	552	linear
KJ128925.1	*Tinca tinca*	*Tinca tinca voucher NRM:54985 16S ribosomal RNA gene, partial sequence; mitochondrial*	552	linear
JF812096.1	*Tinca tinca*	*Tinca tinca voucher PLFRS-24 16S ribosomal RNA gene, partial sequence; tRNA-Leu gene, complete sequence; NADH dehydrogenase subunit 1 (ND1) gene, complete cds; tRNA-Ile and tRNA-Gln genes, complete sequence; and tRNA-Met gene, partial sequence; mitochondrial*	1971	linear

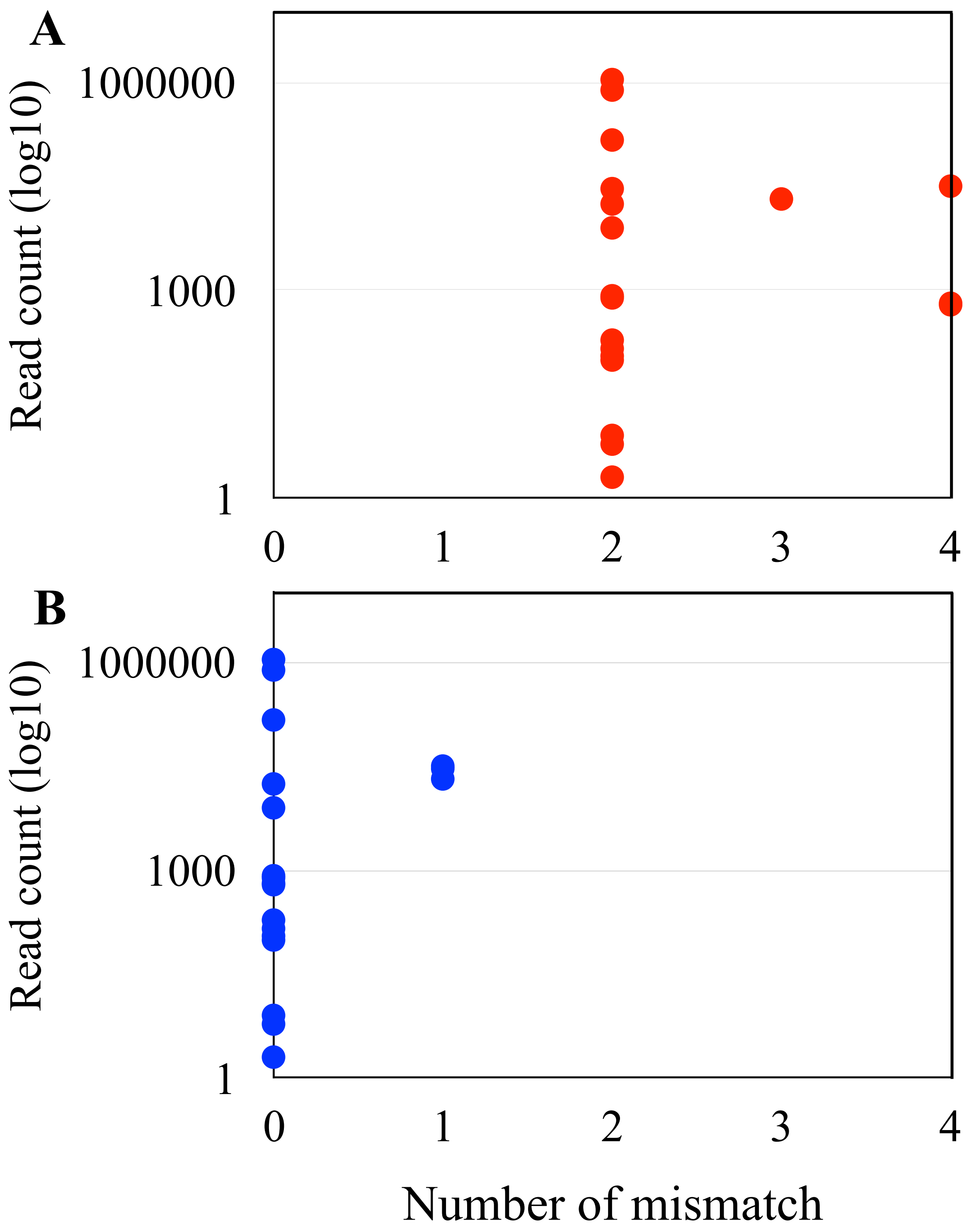

Supplemental Figure S1. Valuation of primers

The correlation of the number of mismatches on primer and read count for A) primer forward and B) reverse.

Supplemental information

Bioinformatic analysis

Raw data from DNA metabarcondig analysis on DNA extracted from excrements of the Eurasian otter (*Lutra lutra*). **Pool 1:** 41_S1_R1_001.fastq.gz/41_S1_R2_001.fastq.gz; **Pool 2:** 42_S2_R1_001.fastq.gz/42_S2_R2_001.fastq.gz; **Pool 3:** 43_S3_R1_001.fastq.gz/43_S3_R2_001.fastq.gz; **Pool 4:** 44_S4_R1_001.fastq.gz/44_S4_R2_001.fastq.gz; **Pool 5**: 45_S5_R1_001.fastq.gz/45_S5_R2_001.fastq.gz.

All sequences were deposited in the ENA's Sequence Read Archive (http://www.ebi.ac.uk/ena) under the accession number PRJEB38720, in FigShare at https://figshare.com/s/10ed08094e13c539e766 and in Dryad Digital Repository at https://datadryad.org/stash/dataset/doi:10.5061/dryad.2bvq83bn5.
